# The Fire-Walker’s High: Affect and Physiological Responses in an Extreme Collective Ritual

**DOI:** 10.1371/journal.pone.0088355

**Published:** 2014-02-20

**Authors:** Ronald Fischer, Dimitris Xygalatas, Panagiotis Mitkidis, Paul Reddish, Penny Tok, Ivana Konvalinka, Joseph Bulbulia

**Affiliations:** 1 Centre for Applied Cross-Cultural Research & School of Psychology, Victoria University Wellington, Wellington, New Zealand; 2 LEVYNA Laboratory for the Experimental Research of Religion, Masaryk University, Brno, Czech Republic; 3 Interactive Minds Centre (IMC), Department of Culture and Society, Aarhus, Aarhus University, Aarhus, Denmark; 4 Center for Advanced Hindsight, Social Science Research Institute, Duke University, Durham, North Carolina, United States of America; 5 Interdisciplinary Centre for Organizational Architecture, School of Business and Social Sciences, Aarhus University, Aarhus, Denmark; 6 Cognitive Systems, Department of Applied Mathematics and Computer Science, Technical University of Denmark, Kgs. Lyngby, Denmark; 7 Department of Cognitive Science, Central European University, Budapest, Hungary; 8 Faculty of Humanities and Social Studies, Victoria University Wellington, Wellington, New Zealand; University of Queensland, Australia

## Abstract

How do people feel during extreme collective rituals? Despite longstanding speculation, few studies have attempted to quantify ritual experiences. Using a novel pre/post design, we quantified physiological fluctuations (heart rates) and self-reported affective states from a collective fire-walking ritual in a Mauritian Hindu community. Specifically, we compared changes in levels of happiness, fatigue, and heart rate reactivity among high-ordeal participants (fire-walkers), low-ordeal participants (non-fire-walking participants with familial bonds to fire-walkers) and spectators (unrelated/unknown to the fire-walkers). We observed that fire-walkers experienced the highest increase in heart rate and reported greater happiness post-ritual compared to low-ordeal participants and spectators. Low-ordeal participants reported increased fatigue after the ritual compared to both fire-walkers and spectators, suggesting empathetic identification effects. Thus, witnessing the ritualistic suffering of loved ones may be more exhausting than experiencing suffering oneself. The findings demonstrate that the level of ritual involvement is important for shaping affective responses to collective rituals. Enduring a ritual ordeal is associated with greater happiness, whereas observing a loved-one endure a ritual ordeal is associated with greater fatigue post-ritual.

## Introduction

Extreme collective rituals have fascinated observers for centuries. Communities worldwide engage in costly and dangerous activities such as body-piercing, flagellation, and fire-walking. Such rituals are not outliers that exist in only a few communities, but are practiced by millions of people around the world. Why? Social scientists have long speculated that participating in extreme collective rituals leads to a state of collective effervescence [1], a shared emotional experience that binds communities together. For example, Haidt [2], [3] argued that one way for individuals to experience intense happiness is to lose themselves in collective rituals, emphasizing the positive affective aspects of these events. Yet other cognitive and evolutionary theorists hypothesize that rituals may invoke both positive and negative affective states, with the latter being particularly powerful in eliciting in-group cohesion and coordination, especially in traditional societies [4]–[7] (for empirical tests see [8], [9]).

Against effervescence models, the apparent physical and psychological hardships endured by participants at extreme rituals would suggest experiences of increased fatigue and discontent [10], [11]. Walking barefoot long distances without consuming water or food, getting pierced with needles and skewers and walking over swords or fire appear painful and exhausting from an outside perspective. Furthermore, it is arguable that witnessing such apparent suffering might plausibly evoke negative affective states, leading to experiences of what might be termed “empathetic exhaustion.” In addition, this empathetic exhaustion might be distributed differentially among spectators, depending on their involvement in the ritual as well as their relationship to the participants [8]. Given these different theoretical models and plausible hypotheses for ritual effects, it is remarkable that that are so few quantitative tests (for exceptions see [8], [9]), with previous studies being largely limited to qualitative, interpretative accounts [12]–[14]. To the best of our knowledge, no research has examined how participants and spectators report their affective reactions to extreme rituals.

To fill the gap, we investigated self-reports on affective states of performers and spectators in a naturally occurring extreme ritual – quantifying emotional states that would be very difficult to simulate in controlled laboratory settings. We did so because observations and attributions based on the nature of the ordeals suggesting that participants in extreme ritual are fatigued and dysphoric may be incorrect. Furthermore, empathetic reactions among ritual participants and observers require more empirical attention. For example, witnessing extreme ritual performances by close kin may induce greater suffering than actually performing the ritual. We used a novel pre-post design to compare participants performing high-ordeal behaviours (body piercings and fire-walking) with participants that accompany fire-walkers but only engage in relatively low-ordeal behaviours (fasting, walking barefoot). Both groups were contrasted with a control group of spectators who were not actively involved in the ritual and were merely watching the central part of the event (the fire-walk).

Affect and Extreme Ritual in Ethnographic and Experimental Research Questions about how participants in collective rituals perceive themselves and the psychological effects of extreme rituals on participants in particular have a relatively long history. Emile Durkheim [1] coined the term ‘collective effervescence’ when describing accounts of village rituals among Australian aborigines: “The very act of congregating is an exceptionally powerful stimulant. Once the individuals are gathered together, a sort of electricity is generated from their closeness and quickly launches them to an extraordinary height of exaltation. Every emotion expressed resonates without interference in consciousness that are wide open to external impressions, each echoing the others” (p 217–218). Contemporary incarnations of this idea include Jonathan Haidt’s [2], [3] hive hypothesis, which argues that the immersion of individuals in large collective events creates one source of strong happiness and well-being. To date, only few controlled laboratory or field studies have investigated affective dynamics among participants in rituals. Simulating the behavioural synchrony that commonly occurs in collective rituals, laboratory-based studies have not found changes in mood or affect after engaging in synchronized group tasks [15]–[17]. In a field study, Konvalinka et al. [8] found support for collective effervescence in the alignment of physiological states among Spanish fire-walkers and related spectators. Extending these results, we compared physiological responses and self-reported affect among groups that differed in ritual involvement.

### Research Context

We examined a religious ritual celebrated annually in a Hindu community in Mauritius, a small island nation in the Mascarene Archipelago, off the East coast of Madagascar. The Thimithi festival is widely celebrated in honour of the Mother Goddess, who takes the form of various deities depending on the local temples and traditions. In the current study, the ritual was devoted to Kali and performed at the village of Pointe aux Piments. The festival lasts ten days consisting of fasting and prayers and culminates in a long procession and a fire-walking ritual. On the day of the fire-walk, people gather in a neighboring village bringing flowers and food offerings to the Goddess. Several devotees engage in body piercings that can vary from needles through the tongue and forehead to skewers 1–2 cm in diameter through the cheeks. After the preparatory prayers, a procession departs for the temple. Participants walk for several hours barefoot on the burning asphalt in the mid-afternoon sun without consuming water or food, stopping at every crossroad to perform apotropaic rituals to ward off evil spirits. Many individuals appear to experience trance-like states during the procession. Upon reaching the temple, fire-walkers walk on the edges of swords before ending their procession by walking over a bed of glowing charcoal. Low-ordeal participants who joined fire-walkers for the procession, as well as other spectators who did not follow the procession, are positioned on the sides of the fire pit. The festival concludes with a common meal for all attendees. This ritual was chosen because it allowed for testing of affective reactions to extreme collective rituals, in a natural setting that is a variant of a larger number of rituals common in religions around the world [5], [18].

### The Present Study

We examined the effects of active and passive participation in this ritual on self-reported affect and physiological response, quantified as heart-rate reactivity. To study these effects scientifically, we used a quasi-experimental pre-post test design [19]. For the affective reactions, we administered a short survey featuring two key affective terms before the beginning of the festival and after the completion of the final event, the fire-walk.

Affect can be differentiated by valence (pleasant – unpleasant) and arousal (activation – deactivation) [20]. Measuring self-reports of affect in a sacred and high-intensity ritual with a semi-literate population presented a challenge. After extensive piloting and discussions with local focus groups, we decided to obtain self-ratings of two states, ‘happiness’ and ‘fatigue’. We focused on ‘happiness’ as an affect term of high positive valence [17], [20]. ‘Fatigue’ was chosen because it is the most deactivated term available in the semantic structure of affect model [20], [21]. The time and logistical constraints of conducting research within the context of a naturally occurring ritual did not allow us to measure affect more comprehensively using established methods. As a physiological measure of arousal (e.g., bodily activation), we measured relative changes in heart rates during the fire-walk compared to the rest of the ritual.

To examine the effects of ritualistic involvement, we sampled fire-walkers (high-ordeal participants); people participating in the ritual without engaging in the high-intensity activities (low-ordeal participants); and spectators (who were neither biologically nor socially related to participants and only observed the fire-walk). The fire-walkers and low-ordeal participants were related through kinship. Low-ordeal participants played a special function in the ritual by providing support for the fire-walkers (e.g., during the piercings of the fire-walkers and the procession by holding and physically supporting the high-ordeal participant, offering special prayers as well as chanting and singing to motivate the high-ordeal participant). The spectators did not take part in any form in the ritual.

The ritual imposes many physical burdens. Participants walked barefoot in the midday sun without eating or drinking while carrying pots of sacrificial offerings (coconuts, milk, food, hot coals). High-ordeal participants were pierced with needles or skewers and finished the procession by walking over knives and then burning coals. Given the strain of these activities on the human body over a prolonged period [22], we expected greater levels of fatigue and lower levels of happiness of participants compared to spectators post-ritual (Hypothesis 1: ritual exhaustion). Specifically, we predicted:

#### Hypothesis 1

There is a decrease in happiness and an increase in fatigue post-ritual moving from high-ordeal participants to low-ordeal participants to spectators.

Alternatively, Durkheim [1] originally speculated that participating in collective events may create states of collective effervescence in a community in general, which in turn may lead to higher levels of happiness post-ritual [2], [3]. It is difficult to operationalize collective effervescence. Interpreting the notion of ‘electricity’ in Durkheim’s writing as potential increases in autonomous activation [6], we predict that heart rates increase during the focal event of the ritual (the fire-walk) compared to the rest of the ritual (which involved physical activities such as walking, indicating an increase over and above physical activation, see [8]). Given the different levels of emotional involvement, we also predicted a linear increase of heart-rates from spectators to low-ordeal to high-ordeal participants.

#### Hypothesis 2

Heart rates will increase linearly with the ritual involvement in the groups, with highest levels of heart-rate increases among fire-walkers.

Moving to self-reported affect, there are a number of mechanisms that can explain positive affect in response to participating in extreme rituals. High intensity performances lasting several hours likely result in release of endogenous opioids [23], [24]. Biological mechanisms of opioid releases during strenuous performance may modulate the experience of affect after the ritual, leading to differentiation of the high and low-ordeal participants, which in turn should show differentiation from mere spectators. Similarly, fire-walkers experience stimuli (body piercings, walking over hot coals) that induce pain, whereas kin-related low-ordeal participants may experience empathy related suffering [8], [9]. Aversive states may trigger opposing, positive reactions when the aversive state is lifted [25], with the relief of pain inducing feelings of pleasure (We would like to thank the editor and one anonymous reviewer for suggesting this mechanism). In line with this theorizing, experimental research has shown that the off-set (removal) of pain in both fruit flies [26] and humans [27], [28] increases positive affect and reduces negative affect. The off-set of physical pain (for fire-walkers) or empathy-driven emotional pain (kin-related low-ordeal participants) post-ritual may result in increased levels of happiness and lower levels of fatigue after the ritual compared to spectators. Therefore, both opioid release and pain-offset mechanisms suggest linear increases of happiness and decreases of fatigue post-ritual depending on the level of ordeal experienced.

#### Hypothesis 3

Post-ritual, highest levels of happiness are reported by high-ordeal participants, followed by low-ordeal participants and then spectators. Conversely, highest levels of fatigue are reported by spectators, followed by low-ordeal participants and lowest levels are predicted for high-ordeal participants.

The role of low-ordeal participants is interesting because of their relationship to the high-ordeal participants, which may create more complex psychological reactions. For experienced fatigue, high-ordeal participants may experience the emotional ‘high’ upon finishing the fire-walk (i.e. upon completing the focal part of the ritual), whereas low-ordeal participants do not experience this ritual ‘high’, while simultaneously worrying about the well-being of their friends and family (the high-ordeal participants). This combined empathic arousal [8] and lack of an emotional ‘high’ explanation could lead to the highest levels of fatigue post-ritual for low-ordeal participants and no difference between high-ordeal participants and spectators (an emphatic identification hypothesis).

#### Hypothesis 4

At time 2, low-ordeal participants will report the highest level of fatigue compared to both high-ordeal participants and spectators, who do not differ from each other.

## Methods

Participants: A total of 70 individuals (36 males) participated in this study, including 28 fire-walkers (14 males), 22 non-fire-walking low-ordeal participants (11 males) and 20 non-related spectators (11 males). Mean age was 32.64 years (SD = 14.90).

Ethics: Mauritius does not have an ethical board that is responsible for non-medical research. All the procedures in our study are non-invasive and the information provided is of a non-sensitive nature. All measures and procedures described in the current study were approved for an independent study in NZ by the School of Psychology Human Ethics Committee under delegated authority of Victoria University of Wellington’s Human Ethics Committee (*RM019281,* approved 23/04/2012). The NZ committee cannot approve research conducted overseas. As there is no equivalent ethics body in Mauritius, we followed the approved procedures deemed applicable in NZ. In line with approved guidelines, informed consent forms were signed by all participants and written permission was obtained by the temple authorities prior to the study. In addition, we informed local authorities of our research and no objections to the study or the procedures were raised.

Procedures: Of the fire-walkers, 8 males received one or more face- or tongue-piercings and 5 females received one tongue-piercing. None of the low-ordeal participants were pierced during the ritual. Low and high-ordeal participants were recruited before the procession; un-related spectators were recruited at the fire-walking site. They completed the pre-ritual questionnaire (time 1) and were individually fitted with an HR monitor. The monitors were attached to a chest strap worn under their clothes, which was unobtrusive and invisible to others. All monitors were synchronized with a central processing unit (Polar Team2 Pro system). The focal HR data were collected during the 18-minute fire-walk that closed the ceremony. Immediately following the ritual, all participants returned the monitors and completed the post-ritual questionnaire (time 2).

Participants were asked how they felt at that moment (using the terms ‘fatigued’, ‘happy’ along a single line anchored by “not at all” and “very much”). Questions were presented in the local Creole dialect. Responses along this scale were measured to the nearest millimetre, ranging from 0 to 20 cm. HR data were recorded as beat-to-beat intervals and extrapolated to beats-per-minute per second intervals. Ectopic heartbeats were identified and removed prior to the analyses. HR data were standardized (M = .00, SD = 1.00) within each individual. Physiological responses were therefore measured as the change in HR during the fire-walking period compared to a baseline (comprising the rest of the measurement period). Two HR monitors malfunctioned (one for a low-ordeal participant and one for a spectator), hence, we analyzed 68 participants to assess HR changes. To rule out potential gender effects, we analyzed data using a 2×3×2 mixed-effects ANOVA with time as the within-subject variable (2: pre-ritual versus post-ritual) and ritual role (3: fire-walker, low-ordeal participant, unrelated spectator) and gender (2: male, female) as between-subject variables. Considering the relatively small sample size, we interpreted effect sizes as well as significance values. We examined any effect equivalent to a medium effect size (η^2^ = .059) [29].

## Results

**Figure 1:**
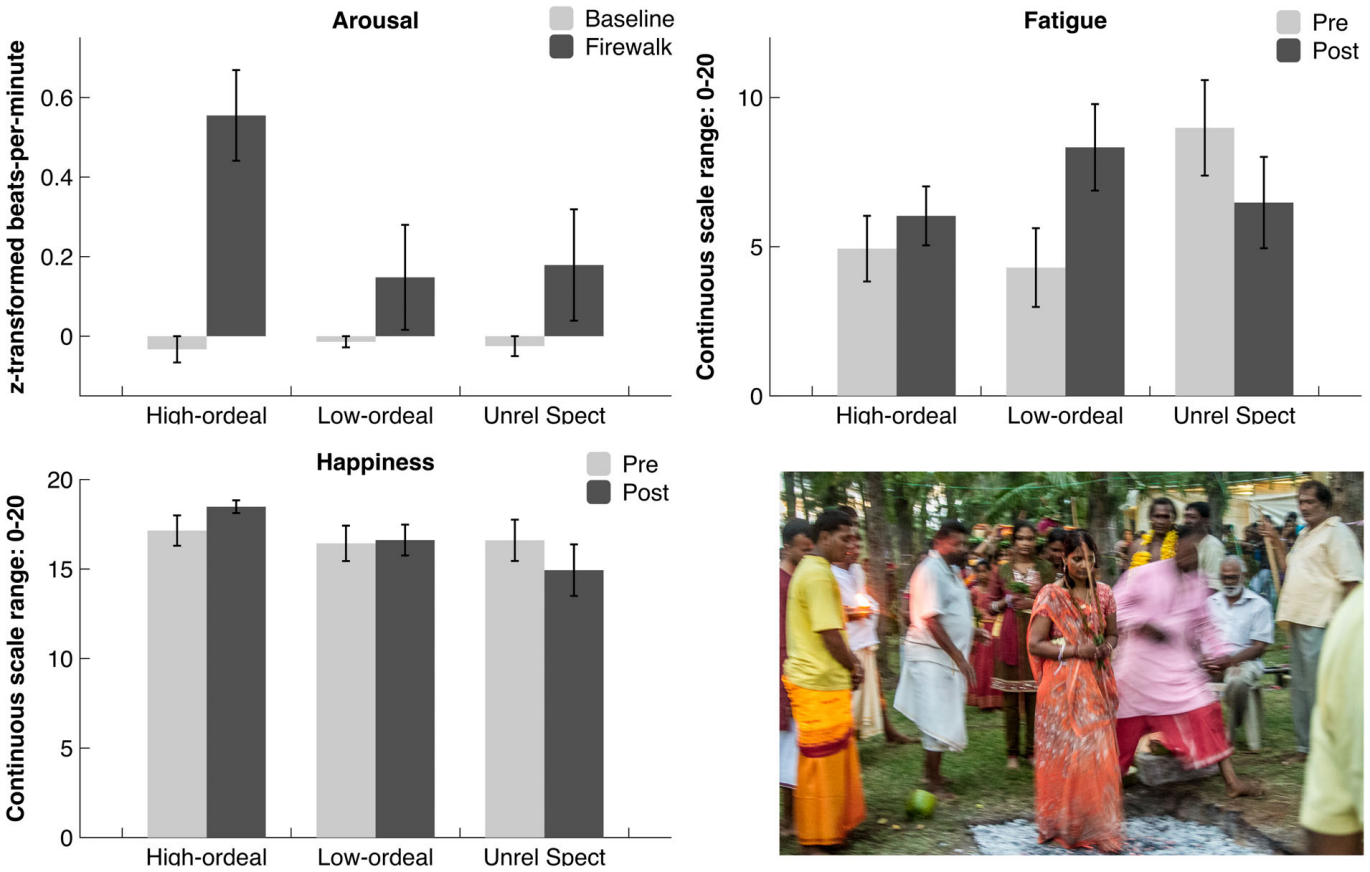
Effects of ritual participation on self-reported affect and heart rates. Upper left panel: Effects of ritual involvement on physiological arousal (z-transformed heart rates measured in beats-per-minute). Lower left panel: Effects of ritual involvement and time on happiness. Upper right panel: Effect of ritual involvement and time on fatigue. Lower right panel: A female fire-walker stepping onto the fire. The individual pictured has given written informed consent (as outlined in PLOS consent form) to publish this picture.

Concerning happiness, we observed a moderate effect size for time×involvement in the ritual: F(2,57) = 2.46, p = .09, partial η^2^ = .080. Breaking down this interaction (see Figure 1), there was no difference in happiness between the groups before the ritual: F(2,60) = .12, p = .88, partial η^2^ = .004, but the groups differed post-ritual: F(2,60) = 4.19, p<.05, partial η^2^ = .123. In line with hypothesis 3, a significant linear trend (p<.01, raw scores and controlling for Time 1 scores) showed that fire-walkers were happiest (M_adjusted_ = 18.48, SE = .82) followed by low-ordeal participants (M_adjusted_ = 16.75, SE = .90) and spectators (M_adjusted_ = 14.65, SE = .93). The changes in happiness within groups were not significantly different between time 1 and 2. Comparing participants to spectators, the interaction was significant: F(1,59) = 4.05, p<.05, partial η^2^ = .064, with high and low-ordeal participants overall reporting higher happiness after the ritual (M_adjusted_ = 18.42, SE = .85) compared to spectators (M_adjusted_ = 15.77, SE = .67). High and low-ordeal participants did not significantly differ from each other. This result supports our hypothesis 3 predicting positive reactions to extreme ritual.

Second, we found the predicted time×ritual involvement interaction for fatigue: F(2,57) = 4.83, p<.05, partial η^2^ = .145. Breaking down this interaction (see Figure 1), we observed a main effect of ritual involvement on time 1 scores: F(2,61) = 4.95, p<.05, partial η^2^ = .140, with spectators experiencing higher fatigue (M = 9.52, SD = 1.35) compared to both high (M = 4.65, SD = 1.15) and low-ordeal participants (M = 4.36, SD = 1.24) (p<.01). At time 2, low-ordeal participants (M_adjusted_ = 9.01, SE = 1.29) experienced higher fatigue compared to spectators (M_adjusted_ = 5.60, SE = 1.39; p = .08) and high-ordeal participants (M_adjusted_ = 6.31, SE = 1.17; p = .12; overall F_T1 adjusted_ (2,63) = 1.85, p = .17, partial η^2^ = .062). In line with empathic identification predictions, the increase in fatigue was largest and significant among low-ordeal participants: t(19) = −3.03, p<.01 (pre-ritual: M = 4.30, SD = 5.90; post-ritual: M = 8.33, SD = 6.48), but not significant among fire-walkers and spectators (t_max = _1.25). Overall, the pattern is in line with hypothesis 4.

Third, there were significant main effects of time: F(1,62) = 15.44, p<.01, partial η^2^ = .199 and ritual involvement on physiological responses overall: F(1,62) = 3.87, p<.05, partial η^2^ = .111, which were qualified by the time by involvement interaction: F(2,62) = 3.17, p<.05, partial η^2^ = .093. The effect of ritual involvement was significant for the fire-walk: F(1,62) = 4.33, p<.05, partial η^2^ = .123, but there was no difference for the baseline responses: F(2,62) = .64, p = .53, partial η^2^ = .020. This supports the collective effervescence hypothesis (Hypothesis 2). The standardized heart rates of the fire-walkers during the fire-walk were significantly higher (M = .59, SD = .57) than that of low-ordeal participants (M = .15, SD = .73) and spectators (M = .19, SD = .44): F_contrast_ (2, 61) = 4.11, p<.05, partial η^2^ = .119. For most fire-walkers, the period of the fire-walk (although the actual fire-walk only lasted a few seconds for each individual) led to much higher heart rate reactivity than the preceding hours of physical activity during the procession and the preparation of the ritual. Spectators and low-ordeal participants did not differ from each other. This only partially confirms our hypothesis 2.

The only gender effect was a significant time×gender interaction for happiness: F(1,57) = 4.53, p<.05, partial η^2^ = .074, with males reporting marginally greater happiness after the ritual (M = 17.05, SD = 3.51) than before the ritual (M = 15.79, SD = 4.84): F(1,61) = 3.43, p<.10, partial η^2^ = .053.

One potential problem with any naturally occurring study like ours is self-selection of participants into the high-ordeal group. We asked all participants how often they have fire-walked before (M = 2.7, range = 0–15) and how often they had watched fire-walks previously (M = 6.9, range = 0–35). We did not observe statistically significant differences between the groups in terms of how often they participated: F(2, 64) = 1.37, p = .26 or how often they had watched a fire-walk: F(2, 62) = .14, p = .87. Therefore, we can rule out self-selection effects due to past experience with the ritual. When controlling for these two variables in our analyses, the results were unchanged in form (the explained variance for the time by condition effect for both self-report variables actually increased slightly).

## Discussion

Our study provides the first quantitative test of self-reported affective reactions to extreme rituals in a field setting. Using a novel quasi-experimental pre-post design, we demonstrated that ritual modulates affective experience, broadly in line with anthropological theories of collective ritual [1], [2]. Happiness linearly increased from spectators to low-ordeal performers and high-ordeal performers after the ritual. Fatigue was highest among low-ordeal performers post-ritual, but high-ordeal participants and spectators did not differ in experienced fatigue after the ritual. Heart rates increased during the fire-walk for all individuals who attended the ritual, but showed the highest activation for fire-walkers. This pattern of performers of the extreme ritual experiencing the highest increase in heart rate and reporting greater happiness after the ritual compared to spectators was inconsistent with Hypothesis 1 predicting negative outcomes of participating in extreme ritual. From an outsider perspective, it may be surprising to see such positive effects after performing what appears to be a very exhausting and dysphoric ritual. Yet, these findings support long-standing theorizing in the anthropological literature that engaging in collective rituals increases positive affect of participants [1, 30, for a study of a less extreme collective ritual, see 11]. More importantly, this study contributes novel data to long-held speculations and ethnographic observations by demonstrating that the affective experience of ritual is shaped by the roles that individuals perform.

### Possible Mechanisms underlying Affect Responses in Extreme Ritual

Supporting hypothesis 3, we found greater happiness among high and low-ordeal participants compared to spectators. Both endogenous release of opioids [23], [24] and off-set of pain mechanisms [25]–[28] may explain this increase in affect post-ritual. The pain-offset mechanism would predict that individuals who engage more frequently in negatively valenced activities will become habituated to the negative states, but will report an increased reversal of the negative state after the end of the activity. To the extent that increased participation in these extreme rituals (i.e. repeated fire-walks) will lead to habituation that increases the positive effects on participants, we should see greater pain tolerance during the fire-walk and increased positive affective reaction after the ritual (asymmetrical reverse) [25]. Similarly, the endogenous release of opioids mechanism suggests that opioids are released with greater efficiency after repeated exposure [23], [24], which may be associated with more positive reactions post-ritual, but also more autonomous nervous system reactivity during the ritual. Both mechanisms are somewhat overlapping. We report some more data that may speak to these explanations and can guide future studies.

Since it was not realistically possible to obtain self-report data during the moment of the fire-walk, we can only compare the pre-ritual to the post-ritual scores. Correlating the frequency of fire-walking with self-report measures and heart rates, no significant correlations emerged. When controlling for age, gender and time 1 (pre-ritual) scores in a regression analysis, the effect of fire-walking experience (number of previous fire-walks) was not significant for fatigue (standardized beta = −.16, p = .50) or happiness (standardized beta = .27, p = .35). More interestingly, habituation effects in the literature tend to be stronger for physiological measures [28]. We did not find significant correlations between heart-rates and fire-walking frequency in our data (r = .26, p = .25), indicating that there was no evidence of habituation in our data. Similarly, no habituation effects of watching fire-walks were found for low-ordeal participants and spectators combined (r = .09, p = .58). It is possible that these annual events may not be sufficient for showing habituation effects, at least with the type of measures that we were able to apply in this field setting. Future studies obtaining self-reports during, as well as before and after events that vary in frequency would be most helpful for a better understanding of the unfolding of affective reactions during extreme ritual.

Supporting hypothesis 4, low-ordeal participants reported significantly higher levels of fatigue following the ritual compared to starting the ritual. This is in contrast to both fire-walkers and spectators, who showed no increase in fatigue from time 1 to time 2. There are two plausible mechanisms that might explain this effect. First, low-ordeal participants did not experience an emotional high after participating in the central part of the ritual (e.g., via release of endogenous opioids). Second, because related participants have family ties to the fire-walkers, they may have subjectively experienced suffering through empathic identification processes [8], [9]. Based on our own ethnographic observations of several extreme rituals as well as interviews with observers, emotional highs may be plausible, especially if the experience is framed in religious terms highlighting the transformative nature of the ritual for all participants and observers (e.g., suffering increasing the blessing for the community; perceived suffering emphasizes the power of religion to transcend worldly ordeal). These emotional reinterpretations require a strong emotional reaction during the ritual, followed by an emotional relief when the ritual ends as well as subsequent additional cognitive efforts to reinterpret these experiences. Therefore, these off-set effects of perceived pain might be less common and harder to demonstrate empirically. The second mechanism of empathetic identification effects is in our view a more likely explanation of the current findings and fits with some of the emerging research on collective ritual. Our data cannot shed further light on these two alternative explanations and more research is needed to differentiate these possible effects.

### Future Research

These findings point to the need for further research into the interactions between biological mechanisms of experiencing affect and the motivations of individuals to participate in extreme rituals. Bastian et al. [31] experimentally demonstrated that individuals who recalled an episode in which they had committed some unethical behavior were more likely to induce pain to themselves compared to people who did not recall such memories. The pain was rated as more intense in the moral digression condition compared to the control condition. Importantly, this experience of pain reduced the experimentally induced guilt compared to a condition where people were not given the option of hurting themselves. Translating these findings to field settings, are individuals who believe that they have transgressed some moral boundaries (committed some sin or are impure in respect to some subjectively or communally salient moral standard) more likely to engage in extreme rituals compared to other individuals? The commonly noted explanation that devotees engage in rituals in order to cleanse themselves from impurity and sin may fit this pattern [31]. This then leads to the central question of our current investigation: Do people who believe that they have transgressed some moral boundaries (committed a sin or have become impure) experience greater pain during the ritual and consequently experience a greater off-set of pain and more positive affect post-ritual? Xygalatas et al. [9] and Olivola and Shafir [32] reported that greater perceived pain resulted in more prosocial behaviour in both field and laboratory settings. We could speculate that the perception of moral transgressions increases the perceived pain among high-ordeal participants in extreme rituals. This perception of pain then leads to a) more prosocial behavior targeted towards the community and b) greater positive affect following the ritual for the individual. The perceived violation of moral standards reinforces the importance of the ritual both for individuals (by providing opportunities for moral cleansing) and the community (by increasing social cooperation and reifying moral standards). The affective responses of individuals therefore connect the individuals and the community back together via psycho-biological experiences of pain during extreme rituals.

The role of spectators is interesting in this regard. High-ordeal participants engage in extreme actions for specific purposes (e.g., moral cleansing, purification, gratitude) [31]. For the high-ordeal participant the purpose of the self-inflicted suffering may justify the pain, leading to no averse effects, whereas for kin the empathetic suffering may not be relieved. The affective responses among related and unrelated spectators of extreme rituals that vary in the level of ordeal for participants are one of the perplexing questions to be answered in future research. Our findings in light of the experimental literature offer some interesting points of departure for future theorizing and research on extreme rituals.

To summarize, this study offers the first quantitative examination of self-reported affective reactions during extreme rituals in a naturalistic setting and provides novel insights and questions about the affective reactions to extreme stimuli. We demonstrate that affective reactions to extreme collective rituals are divergent and are shaped by the roles assumed within the ritual context. Perceiving apparent suffering of people close to oneself can be more distressing and exhausting than suffering oneself.
